# Supplementary material for: The causal relationship between the human gut microbiota and pyogenic arthritis: a Mendelian randomization study
Source: Front Cell Infect Microbiol. 2024 Nov 26;14:1452480. doi: 10.3389/fcimb.2024.1452480 (PMC11629706; doi:10.3389/fcimb.2024.1452480)
Supplement: Supplementary file 1 [file DataSheet1.zip › mendelian test/ebi-a-GCST90027817.csv_leave_one_out.pdf]

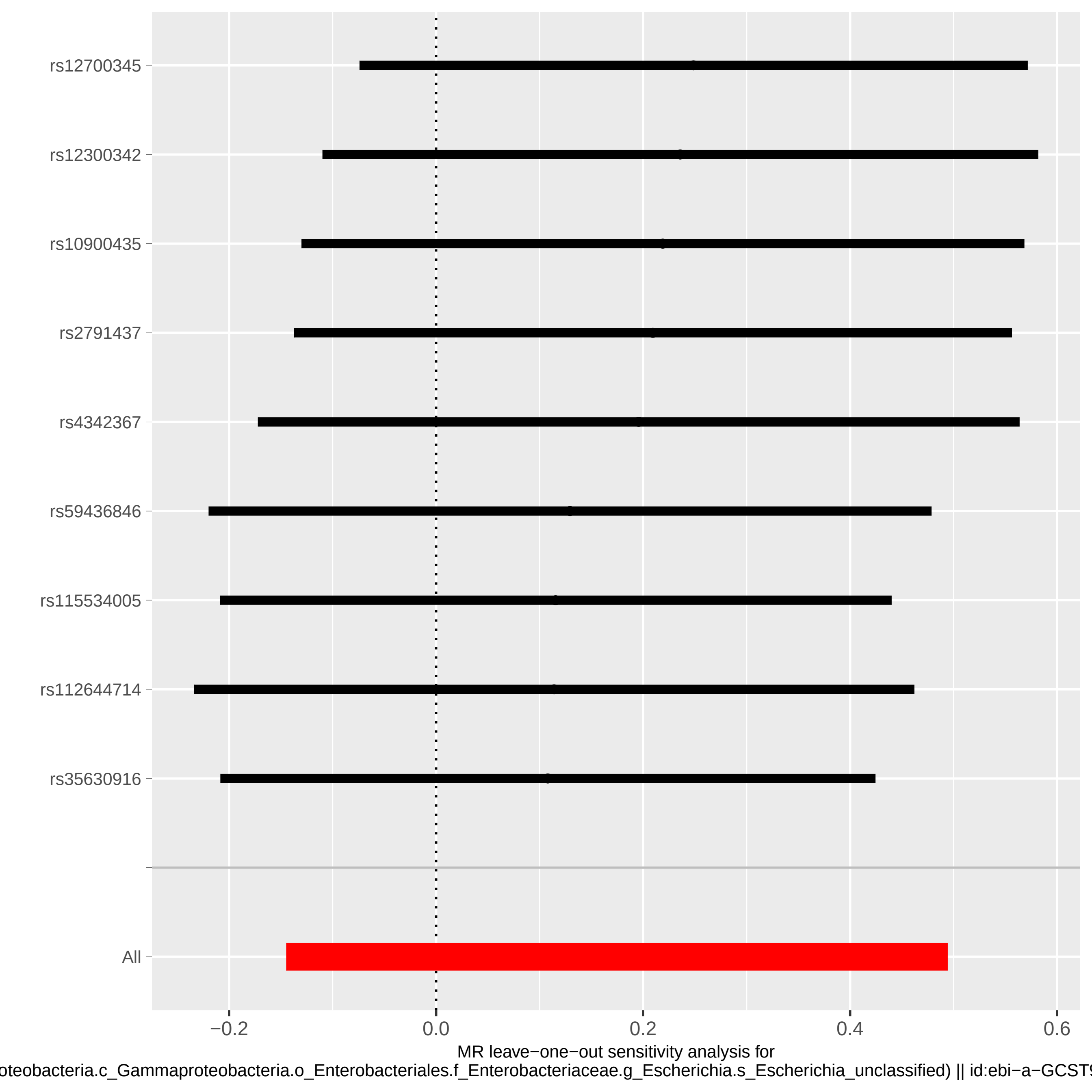
rs12700345
rs12300342
rs10900435
rs2791437
rs4342367
rs59436846
rs115534005
rs112644714
rs35630916
All
−0.2
0.0
0.2
0.4
0.6
MR leave−one−out sensitivity analysis for
oteobacteria.c_Gammaproteobacteria.o_Enterobacteriales.f_Enterobacteriaceae.g_Escherichia.s_Escherichia_unclassified) || id:ebi−a−GCST
